# The Fate of Cervical Dysplastic Lesions during Pregnancy and the Impact of the Delivery Mode: A Review

**DOI:** 10.7759/cureus.42100

**Published:** 2023-07-18

**Authors:** Shirin Dasgupta

**Affiliations:** 1 Dr. B. C. Roy Multi Speciality Medical Research Centre, Indian Institute of Technology, Kharagpur, IND

**Keywords:** postpartum regression, rate of progression, mode of delivery, cervical intraepithelial neoplasia, cervical dysplasia

## Abstract

Cervical dysplasia, also referred to as cervical intraepithelial neoplasia (CIN) or squamous intraepithelial lesion (SIL), is the precursor lesion of cervical carcinoma. Therefore, its diagnosis is vital for early detection and inhibiting the development of cervical carcinogenesis. Human papillomavirus (HPV) is the most common aetiology of cervical cancer and this infection mainly affects young women of childbearing age, thus affecting pregnant women as well. It is essential to know how CIN progresses in pregnant patients because the management of pregnant and non-pregnant patients is different (considering the safety of both mother and child in pregnancy). This review intends to highlight the studies which have assessed the rates of progression of CIN diagnosed in pregnancy throughout the antenatal period and the impact of the mode of delivery on CIN outcomes. We searched PubMed/MEDLINE and Google Scholar databases for relevant articles. Many studies indicate that the rate of progression of these lesions is very slow during the tenure of pregnancy; many also report postpartum regression of these lesions. Thus, in most of these patients, management can be safely implemented in the postpartum period while just keeping them under observation in the antenatal period. However, patients with high-grade CIN have a higher chance of developing invasive cancer and, therefore, require careful monitoring. There is a dispute regarding the role of the mode of delivery in determining the fate of cervical dysplasia. While some studies supported vaginal births over caesarean sections, others did not find any difference between the two in defining the outcome of the dysplastic lesions.

## Introduction and background

In 2018, cervical cancer was categorised as the fourth most common cancer prevailing among women worldwide. The estimated age-standardized incidence was 13.1 per 100,000 women with a huge interregional inconsistency. Cervical cancer was also responsible for over 311 million deaths globally during the same year, thus becoming the fourth leading cause of mortality among women. Medium- and low-resource countries face the highest mortality [1]. In India, cervical cancer is a major health problem. There are differences in the range of diseases affecting cervical health in the rural population when compared to urban areas owing to various factors based on the education, attitude, and awareness of the population [2]. Cervical dysplasia, also referred to as cervical intraepithelial neoplasia (CIN) or squamous intraepithelial lesion (SIL), is the precancerous stage of cervical cancer. It can be low-grade or high-grade; the screening methods aim to detect these lesions in order to prevent the development of cervical carcinogenesis.

The commonest aetiology of cervical cancer is high-risk human papillomavirus (HPV) type 16 and 18, which has the highest oncogenic potential [3]. Cervical cancer has also been recognised to have a long pre-malignant stage; therefore, timely intervention will aid in early diagnosis and prevent cervical carcinogenesis. A cervical Pap smear studies the cytology of the cervical cells collected from patients; this test can aptly diagnose cervical dysplastic and premalignant lesions, alerting clinicians to follow appropriate treatment plans accordingly.

In pregnant women, the prevalence of abnormal findings in cervical cytology is about 2-7%, which is almost the same as that of the non-pregnant population [4]. About 1-3% of cervical cancer cases are diagnosed during pregnancy or the postpartum period [5]. CIN is predominant in women aged 20-34 years [6], which is pretty much the childbearing age across most geographical locations. Because this age group presents the highest frequency of HPV infections and cervical dysplastic lesions, cervical cancer is the most common malignancy during pregnancy [7].

A review article states that the peak incidence of CIN and pregnancy co-existence occurs during the third decade of life [8]. Although there are no specific guidelines for cytological screening in pregnancy, prenatal cervical cytology is a strong platform for detecting dysplastic lesions. Abnormal cytology is common in pregnancy. Although some pregnancy-related changes may alter the cytological microscopy findings, cervical cytology (Pap smear) is still considered a standard screening tool. In pregnant women, CIN is diagnosed 100 times more frequently than invasive cancer [9].

Colposcopy is an essential tool for evaluating cervical dysplasia during pregnancy. The American Society for Colposcopy and Cervical Pathology recommend colposcopy during pregnancy and postpartum in patients with an immediate risk of high-grade CIN of more than 4% [10]. However, colposcopy in pregnant patients is more challenging compared to the non-pregnant counterparts as pregnancy-induced hormonal variations result in cervical epithelial hyperaemia, endocervical gland enlargement, overproduction of mucus, contact bleeding, and prolapsed vaginal walls [11]. Therefore colposcopy must be performed only by a skilled clinician.

Patsner et al. conducted a study to analyse the fate of low-grade CIN during pregnancy [12]. It was concluded that pregnant patients who had biopsy-proven mild cervical dysplasia with expert colposcopy and Pap smear results consistent with biopsy, require only one antepartum colposcopy prior to postpartum revaluations and treatment. Numerous cytological and colposcopic studies with or without biopsy are needless. Also, the odds of these patients developing invasive cancer are meagre.

The supervision of pregnant women with atypical findings in cervical cytology is clinically challenging. While, the health of the mother cannot be compromised, and invasive lesions (if any) must be excluded, the unborn baby also should not be made vulnerable to any preventable risks [6].

Throughout the past few years, there have been many studies pertaining to the outcome and progression of CIN or cervical dysplastic lesions throughout pregnancy, whether they progressed, remained persistent or regressed, and the results obtained are heterogeneous. Also, whether the mode of delivery (caesarean section vs. vaginal delivery) affects the outcome of these cervical lesions has been under constant supervision and research. While some authors advocate vaginal delivery as a safer option in pregnant patients with CIN [13-16], others have found no such correlation between the mode of delivery and the progression of CIN [17,18].

This review will highlight the salient features of some studies that focus on the rate of progression, persistence, or regression of cervical dysplastic lesions diagnosed in pregnancy. It shall also put forward facts that had been inferred about the role of the mode of delivery affecting the outcome of cervical dysplastic lesions in pregnancy.

## Review

Methodology

PubMed/MEDLINE and Google Scholar databases were searched with the keywords “Cervical dysplasia”, “Pregnancy”, “Progression”, “Mode of delivery” and others. About 100 publications including original research and review articles pertaining to this topic were reviewed.

The outcome of cervical premalignant/dysplastic lesions in the antenatal period

The natural history of CIN in pregnancy has been subject to conflicting reports. Origoni et al. stated that the rate of progression of CIN to invasive cervical cancer is 0.4%, which is extremely low [19]. On the other hand, Coppolillo et al. detected a higher rate of progression, of about 13.3% [20].

Spontaneous regression in the postpartum period has been reported to be in the range of 16.7-69.3% among pregnant women with high-grade CIN [21]. There is a theory that sex hormones overexpress themselves during pregnancy and thus facilitate cervical carcinogenesis by initiating transformation zone squamous metaplasia as well as local immune system modification. The sex hormones reduce in level post-delivery; thus exhibiting a possible explanation for the augmented regression in that period [22].

Coppola et al. conducted a retrospective study on pregnant patients diagnosed with high-grade CIN to determine the outcome of the lesions in these patients [23]. The regression rate was as low as 12%. It was concluded that there is a high rate of persistence and progression of the disease due to its underdiagnosis by cervical cytology and therefore supports the fact that invasive biopsy should be done regularly in these patients.

Palle et al. conducted a retrospective study to assess the fate of CIN during pregnancy [24]. This study was carried out in pregnant patients with abnormal cytology reports. It was reported that there was 75% persistence or progression of CINs in the post-partum period. It was concluded that due to the high persistence rate of CIN in pregnancy, generous use of colposcopy-guided biopsies is recommended during pregnancy and a considerable follow-up rate in the postpartum period should be guaranteed.

Kaplan et al. carried out a retrospective study to assess the prognosis and recurrence risk for patients who were diagnosed with CIN in the antepartum period [25]. In this study, 32% of patients with antepartum findings of low-grade CIN on a Pap smear had persistent low-grade CIN during the postpartum period, 6% progressed to high-grade CIN, and the rest regressed spontaneously. On the contrary, cases diagnosed as high-grade CIN persisted during pregnancy and into the postpartum period and 11% progressed to invasive carcinoma. Therefore, most of the low-grade lesions regressed whereas the high-grade CINs persisted and even progressed to invasive carcinoma.

A retrospective study of abnormal Pap smears of pregnant patients to study the post-partum progression or regression of cervical dysplasia diagnosed during pregnancy observed that there was a low progression rate of cervical dysplasia during pregnancy and regression rates postpartum were high (63-76%) for all the lesions of cervical dysplasia [26]. So, conservative therapy throughout pregnancy, as well as postpartum follow-up, is suggested.

In 2006, the American Society of Colposcopy and Cervical Pathology (ASCCP) published revised guidelines for the management of abnormal cervical cytology and histologic lesions in pregnant and non-pregnant women [14,27,28]. They stated that in pregnant patients who had atypical squamous cells (ASC) or low-grade CIN diagnosed in cytology, colposcopy as well as subsequent management measures could be postponed up to six weeks postpartum, because these abnormalities were likely to regress spontaneously and progress to invasive malignancy.

Fader et al. carried out a retrospective study to investigate the fate of dysplastic cervical lesions diagnosed through cytology in pregnant patients in relation to antepartum colposcopy and cervical biopsy [29]. It was the largest study until then and included 1079 pregnant patients. Cytology of the pregnant patients showed 30% were diagnosed with ASC, 55% were diagnosed with low-grade CIN, and 15% were diagnosed with high-grade CIN. Colposcopic examination correlated well with the cytological diagnosis and also with subsequent cervical biopsy even though biopsy was performed infrequently. The authors concluded that there was a regression of cervical dysplasia in the postpartum period; therefore, there was no progression to invasive cancer during pregnancy. Colposcopy and biopsy were unessential as they correlated well with cytology. This study supported the statement of the ASCCP laid down in 2006 because 86% of the low-grade lesions in their cohort regressed back in the post-partum period.

Rueckert et al. carried out a case-control study of pregnant women with severe cervical dysplasia as well as non-pregnant controls to relate postpartum regression and progression rates [30]. They supported the thesis that non-surgical management in pregnant patients is harmless because no progression to cervical cancer occurred in them, thus concluding that the conservative management of pregnant patients with high-grade cervical dysplasia yielded excellent results. Therefore, one could even consider waiting longer than 8-12 weeks postpartum prior to executing a surgical plan.

Gomez et al. carried out a study to analyse the progression of high-grade CIN through pregnancy as well as the postpartum period [31]. This study also aimed to define factors associated with the regression of dysplasia. They stated that a high-grade CIN diagnosis on cervical Pap smear in the third trimester of pregnancy was an independent predictive factor of the persistence of high-grade dysplasia postpartum. This study reported a 43% regression rate of high-grade CIN and there were no cases of invasive lesions at postpartum follow-up. Performing cervical cytology in the third trimester shall help identify patients at risk of persistent CIN after delivery.

Surgical intervention of the cervix during pregnancy has been reported to be associated with preterm births [32-35]. However, Lantsman et al. stated in their study that surgical intervention performed during pregnancy in cases of cervical dysplasia were not associated with pregnancy complications [36].

Korenaga and Tewari in their review article recognised cervical cancer as the most common gynaecological malignancy diagnosed in pregnancy [8]. During pregnancy, abnormal cytology is common and can account for up to 5% of tests. In women who are immunocompetent, CIN seldom progresses to invasive disease during pregnancy.

Pereira et al. in an observational analytical and retrospective study in Brazil did not find significant differences between pregnant and non-pregnant women, with respect to the occurrence of precursor lesions of cervical cancer. Thus pregnant women have the same risk as non-pregnant women in presenting with cervical dysplastic lesions [7].

Another study obtained postpartum CIN regression rates ranging from 37% to 74%, with regression of low-grade CIN in about 80% of cases [13]. Cervical dysplasia regresses once the temporary immunosuppressive state of pregnancy subsides.

Freudenreich et al. assessed the course of dysplasia during pregnancy (the rate of persistence and remission) and opined that attentive monitoring of CIN during the antenatal period is sufficient and clinically safe [6]. Postponing the therapy or treatment for CIN to the postpartum period did not seem to pose any harm to mother or child.

Suchońska et al. conducted a study to assess the natural history of CIN during pregnancy and postpartum period and observed that several CIN cases tend to spontaneously regress following delivery [9].

Stuebs et al. evaluated the progress of untreated high-grade CIN during pregnancy and measured progression, persistence, and regression in the postpartum period [11]. Their study concludes that conservative management is safe for women with high-grade CIN during pregnancy as the rate of progression is slow, but cautious postpartum evaluation is necessary.

Literature reveals that pregnancy and postpartum period witness higher remission rates of CIN than non‑pregnant states. Following delivery, a considerably higher rate of spontaneous remission of even high intraepithelial lesions is seen while progression or persistence of CIN is infrequent.

The reason for the regression of cervical intraepithelial dysplastic lesions after delivery as observed in many studies may be due to the immunosuppressive effect of sex hormones during pregnancy.. On biopsy, the dysplastic epithelium may be excised in its entirety. In addition, disruption of the epithelial integrity during delivery may trigger a local immune response leading to the loss of dysplastic tissue [17,20].

The impact of the mode of delivery on the progression of cervical dysplasia

The association of delivery mode with the progression of CIN in pregnant patients is a hazy field of research to date. A pathophysiological theory was considered that the injury as well as the transitory ischemia of the cervix caused during the final stages of labour lead to a tough inflammatory response, thus promoting repair mechanisms and cervical remodelling through the local build-up of inflammatory cells [19]. Additionally, dysplastic foci are lost during cervical ripening and foetal passage through the birth canal. This mechanism is also thought to contribute to the regression of CIN [21,37] Supporters of vaginal delivery debate that this loss of the dysplastic epithelium as well as the passage of the foetus may be another possible reason responsible for partial or total remission of dysplasia post vaginal delivery [13-16]. However, the rates of CIN regression put forward by Yost et al. [17] and Cubo-Abert et al. [18] do not show considerable differences between patients who faced a vaginal delivery and those who underwent caesarean section. All these theories are yet to be confirmed.

Schuster et al. did not observe any difference in CIN regression rate among the two groups of patients with abnormal cervical cytology diagnosed during pregnancy; the two groups being patients with vaginal delivery and caesarean section, thus indicating that the impact route of delivery does not influence the fate of the dysplastic lesions [38]. On the contrary, a retrospective review carried out by Chung et al. stated a 92.9% rate of regression in women with vaginal delivery and 63.2% in women who had caesarean section [39]. Also, persistence rate of lesions (15.8%) was more for the caesarean section group than the vaginal delivery (2.4%). The rate of progression of CIN was also more for patients who undertook caesarean section (21.1%) compared to those who had undergone vaginal delivery (4.8%).

Adhoot et al. studied whether pregnant women with abnormal cervical cytological findings (dysplasia) in antepartum differ in their postpartum rates of regression, taking into consideration the mode of delivery [40]. The cervical cytology (Pap smear) reports of these patients were collected during the antepartum and postpartum periods and then the disease progression was noted. It was reported that there was a higher rate of regression of cervical dysplasia in women who underwent vaginal delivery when compared to those who had caesarean section.

On the other hand, Coppola et al. did not find any such association between mode of delivery and rate of cervical dysplasia [23]. Kaneshiro et al. too concluded in a study that the mode of delivery does not impact the natural history and progression of cervical dysplastic lesions [16].

Bracic et al. conducted a retrospective study to evaluate the fate of CIN in pregnancy and the influence of delivery mode on the outcome [4]. This study concluded that the mode of delivery does not influence the development of CIN and thus should not influence the individual birth plan. During pregnancy, cervical dysplasia showed high rates of persistence and regression. These lesions rarely progress to invasive disease, thus conservative management in pregnancy with postpartum follow-up is a safe protocol regardless of the mode of delivery.

Korenaga and Tewari, in their review article, stated that higher regression rates of CIN have been described with vaginal delivery (60-66%) when compared to caesarean (12%), probably through debridement of dysplastic focus by vaginal birth trauma [8].

Douligeris et al. carried out a meta-analysis aimed at assessing the effect of the mode of delivery on the natural evolution of cervical dysplastic lesions in pregnant patients [5]. This study concluded that the mode of delivery does not amend the natural progression of CIN in pregnancy; thus the presence of cervical dysplastic lesions should not regulate the mode of delivery in these patients.

According to a study by Stuebs et al., the rate of regression of cervical dysplastic lesions was nearly two times higher for vaginal delivery (27.4%) when compared to caesarean sections (15.2%) [11]. Also, a higher progression rate was detected in women who undertook caesarean sections (6.5%) in comparison to those who faced vaginal delivery (2.7%).

Therefore, the impact of the mode of delivery in determining the fate of cervical dysplastic lesions is a topic which is still in debate and further studies involving larger cohorts and different topographical locations should be considered before shedding further light into this topic.

## Conclusions

A cervical Pap smear is compulsory during pregnancy as early as possible (preferably in the first prenatal visit itself). This will help in detecting CIN (if present) early. The treatment for cervical dysplastic lesions diagnosed in pregnancy can be deferred to the post-partum period and this has been found to be safe for both mother and child. These lesions have very low rates of progression and high rates of persistence during pregnancy. Although some studies have also highlighted that cervical dysplastic lesions tend to regress spontaneously after delivery, one should not expect such outcomes from all patients. Thus the patients must be carefully evaluated in the antenatal and postpartum monitoring and careful follow-up are essential. Patients with CIN (especially high-grade CIN) who miss their scheduled follow‑up visits are at the highest risk of developing cervical cancer.

Although no concrete evidence has been put forward so far regarding a connection with the mode of delivery, few studies opine that vaginal delivery is a safer option for CIN patients as there is disruption of the dysplastic cervical foci during the foetal passage through the vagina. However, there are other studies that do not find any correlation between the mode of delivery and the regression of these lesions. Whether vaginal delivery or caesarean section should be practised in these patients is a question which does not have a definite answer. However, unless a caesarean section is otherwise indicated, it is advisable to go for a vaginal delivery. It is anticipated that the facts put forward in this review will aid future investigators in carrying out further research in this field.
